# Roles of Chinese Medicine and Gut Microbiota in Chronic Constipation

**DOI:** 10.1155/2019/9372563

**Published:** 2019-05-21

**Authors:** Zhenyuan Xu, Tianhao Liu, Qingli Zhou, Jing Chen, Jiali Yuan, Zhongshan Yang

**Affiliations:** ^1^College of Basic Medical Sciences, Yunnan University of Chinese Medicine, 650500 Kunming, Yunnan, China; ^2^Yunnan Key Laboratory of Molecular Biology of Chinese Medicine, 650500 Kunming, Yunnan, China; ^3^College of Chinese Medicine, Jinan University, 510632 Guangzhou, Guangdong, China

## Abstract

Chronic constipation is a common gastrointestinal dysfunction, but its aetiology and pathogenesis are still unclear. Interestingly, the compositions of the gut microbiota in constipation patients and healthy controls are different. Various studies reported the different gut microbiota alterations in constipation patients, but most studies indicated that constipation patients showed the decreased beneficial bacteria and the reduced species richness of gut bacteria. Besides, the alterations in the gut microbiota may lead to constipation and constipation-related symptoms and the regulation of gut microbiota has a positive effect on gut functional diseases such as constipation. Microbial treatment methods, such as probiotics, prebiotics, synbiotics, and fecal microbiota transplantation, can be used to regulate gut microbiota. Increasing evidences have suggested that Chinese medicine (CM) has a good therapeutic effect on chronic constipation. Chinese medicine is well known for its multitarget and multimode effects on diseases as well as less side effects. In previous studies, after the treatment of constipation with CM, the gut microbiota was restored, indicating that the gut microbiota might be the target or important way for CM to exert its efficacy. In this review, we summarized the effects of microbial treatment and CM on the gut microbiota of constipation patients and discussed the relationship between CM and gut microbiota.

## 1. Introduction

Chronic constipation is a common gastrointestinal disorder with prevalence of 5%~20% [1, 2]. Constipation symptoms occur in many diseases [3]. Based on the Rome IV criteria, a typical symptom of chronic constipation is the difficult, infrequent, or inadequate bowel movements [4]. Its pathogenesis process is mainly related to diet type, colon motility, and absorption, genetic susceptibility, and behavioral, biological, and pharmacological factors [5]. In fact, the gut microbiota living in the gastrointestinal tract can help digest food into absorbable nutrients, participate in the host immune system, protect the intestinal mucosal barrier, inhibit the growth of pathogenic bacteria, and produce more important metabolites so as to keep the body healthy. Notably, increasing evidences indicated that gut microbiota was associated with chronic constipation, so more studies had been conducted to treat chronic constipation from the perspective of regulating gut microbiota [6, 7]. It is worth mentioning that CM under the guidance of CM theory also can alleviate constipation [8]. Some Chinese scholars studied the mechanism of CM for chronic constipation treatment from the perspective of gut microbiota. However, the relationship between gut microbiota and chronic constipation or the relationship between CM and constipation-related gut microbiota is still not fully clear. In the paper, we reviewed the role of microbial treatment methods of chronic constipation such as probiotics, prebiotics, synbiotics, and fecal microbiota transplantation and discussed the relationship between CM and gut microbiota from three perspectives of CM holistic concept, CM Yin-Yang balance theory, and CM constitutional theory.

## 2. Alterations of Gut Microbiota in Chronic Constipation

The total quantity of microbiota in our body is estimated to be 100 trillion cells, which is ten times of the quantity of human cells and they can carry 300-fold more unique genes than human genome [9]. The gut microbiota colonizes the human gut for a long time and has a symbiotic relationship with the host. Moreover, the normal microbiota and its metabolites with the host can promote gut digestion and absorption functions, improve host immunity, inhibit the growth and reproduction of pathogenic bacteria, protect the intestinal mucosa, maintain the homeostasis of the gut microenvironment, and thus keep the body healthy. In case of the gut microbial imbalance, gut dysbiosis occurs and displays the low abundance of flora and the prevalence of pathogenic bacteria, thus inducing or aggravating many chronic diseases, including chronic constipation [10].

**Figure 1:**
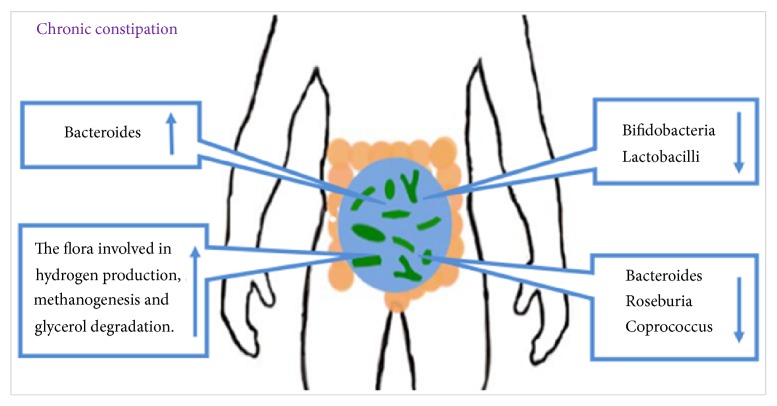
Alterations of gut microbiota in chronic constipation. The gut microbiota is different between constipation and nonconstipation individuals. In constipation individuals, the abundances of* Bacteroides* and the flora involved in hydrogen production, methanogenesis, and glycerol degradation were increased, whereas the abundances of* Bifidobacteria*,* Lactobacilli*,* Bacteroides, Roseburia*, and* Coprococcus* were decreased.

Gut microbiota disorder might be one possible pathophysiological mechanism of constipation [4]. The alterations in gut microbial structure and composition in chronic constipation were reported and the structure and function of the gut microbiota were different between constipation and nonconstipation individuals [7, 11]. For instance, the quantities of* bifidobacteria* and* lactobacilli* in adults with functional constipation were significantly reduced compared with control subjects, whereas the quantity of* Bacteroides *was increased [12]. Mancabelli L et al. [13] found that the gut microbiota of chronic constipation individuals was depleted of the members of* Bacteroides*,* Roseburia, *and* Coprococcus*, but the flora involved in hydrogen production,* methanogenesis*, and* glycerol* degradation exhibited high abundances [14]. However, Ren X et al. [15] found that, at the phylum level, the microbiome of constipation mice showed an increased abundance in the* Firmicutes* and decreased abundances in* Bacteroidetes* and* Proteobacteria*. At the family level,* Lactobacillaceae* and* Bifidobacteriaceae* increased and S24-7 and* Prevotellaceae* decreased in constipated mice.* Prevotella9* was the decreased characteristic bacteria in constipation patients [16] (Figure 1).

## 3. Changes in Gut Microbial Metabolites in Chronic Constipation

Gut microbiota can metabolize substances that the host itself cannot metabolize and participate in host metabolism to generate a series of metabolites, such as cholesterol and bile acid metabolism, and hormones metabolism. Some metabolites play a pivotal role in maintaining the balance of water and electrolytes in the intestine, the normal structure of gut microbiota, and even anti-inflammatory, intestinal function, and immune regulation. Growing evidences indicate that gut microbiota imbalance is closely related to the occurrence of chronic constipation. Therefore, it is necessary to explore the relationship between the changes in gut microbiota metabolites and host diseases for the improvements in chronic constipation prevention and treatment.

**Figure 2:**
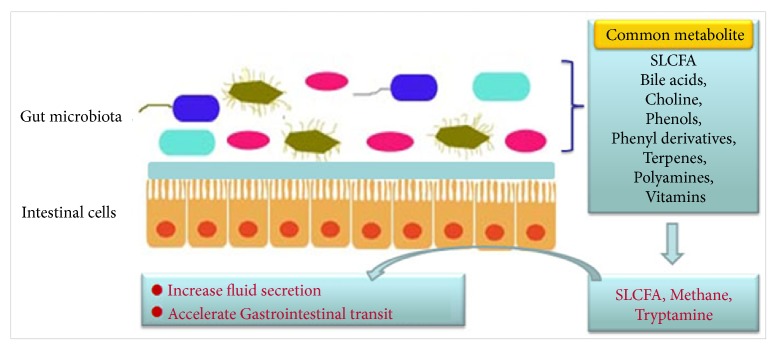
Common metabolites and their effects on the intestines. Particularly SLCFA, methane, and tryptophan increased mucus secretion and accelerated gastrointestinal transit.

As shown in Figure 2, the gut microbiota interacts with the host to produce metabolites such as short-chain fatty acids (SCFA), bile acids, choline metabolites, phenols, phenol derivatives, terpenoids, polyamines, lipids, vitamins, and hormones. Short-chain fatty acids and methane have a direct regulatory effect on gastrointestinal (GI) movement in patients with constipation [17, 18]. However, only a handful of reports indicated possible metabolites associated with chronic constipation, as well as their causal relationships and mechanisms. Bhattarai Y et al. [19] revealed that the bacterial metabolite tryptamine increased anion-dependent fluid secretion and accelerated host gastrointestinal transit via activating the epithelial 5-hydroxytryptamine receptor 4 (5-HT4R) in the proximal colon. Saturated long-chain fatty acid (SLCFA) generated by gut bacteria also enhanced rat colonic contraction and stool frequency [20].

## 4. Microbial Treatment of Chronic Constipation

Recent studies on gut diseases and gut microbiota revealed the relationship between chronic constipation and gut microbiota disturbance, providing a theoretical basis for microbial treatment of chronic constipation. Microbial treatment methods mainly include probiotics, prebiotics, synbiotics, and fecal microbiota transplantation (FMT) [21].

### 4.1. Probiotics

Probiotics, especially* Bifidobacterium lactis*, have a positive impact on shortening overall and regional gut transit time (GTT), increasing stool frequency, and improving stool consistency [22]. After constipation mice were treated with* Bifidobacterium adolescentis *CCFM 669 and 667, the relative abundance of* lactobacilli* increased and the relative abundance of* Clostridia* decreased.* B. adolescentis *CCFM 669 and 667 relieved constipation symptoms by increasing the concentrations of butyric acid and propionic acid and adhering intestinal epithelial cells [23].* Lactobacillus reuteri* (DSM 17938) relieved chronic constipation by significantly decreasing CH_4_ production [14]. Dimidi E et al. [12] also found that modifying the gut luminal environment with certain probiotic strains might affect intestinal motility and secretion, thereby benefiting constipation patients. Changing gut movement by regulating the biosynthesis of serotonin might be one of the mechanisms by which the gut microbiota improved constipation [24]. In addition, probiotics could alleviate constipation and improve other symptoms caused by constipation. For example, probiotics protected neuronal health by activating the protein kinase (AKT) signaling pathway, thereby alleviating depression caused by constipation [25].

However, simple supplementation of probiotics does not necessarily change the human gut mucosal microbiome. Human beings have specific intestinal mucosal colonization resistance to probiotics and personal microbiome and host features are different among different persons [26]. Therefore, probiotics colonization ability in the gut mucosa shows personal differences. The efficacy of probiotics for constipation was not definite. Individual differences in gut microbial characteristics may affect the function of probiotics. For instance, in a study on the effects of probiotics on children with constipation, PEG plus probiotics (*B. breve M-16V*®*, infant M-63*®, and* longum BB536*) did not show the superior treatment efficacy of constipation compared to PEG treatment [27].

### 4.2. Prebiotics

Prebiotics are food ingredients used to boost microbiota in the gastrointestinal tract and their main dietary sources are mushrooms, oats, barley, seaweed, and other fiber-rich foods [28]. It is worth noting that prebiotics can selectively stimulate the growth and activity of beneficial bacteria and that the stimulation may be a vital mechanism for alleviating constipation [29]. Huang P et al. [30] treated postpartum constipation patients with lactulose and found that the improvements in constipation symptoms were significantly better than those in control group, such as increased remission time, prolonged constipation-free days, and decreased defecation time. A common type of edible marine algae Enteromorpha (EP) containing rich carbohydrates, proteins, crude fiber and vitamins, and polysaccharides from canola (PEP) was used to interfere with constipation and then found to reverse intestinal inflammation and recover intestinal function [15]. As a prebiotic, inulin-type fructan showed a selective effect on the human gut microbiota, especially* bifidobacteria*, anaerobic bacteria, and* Bilophila*, and improved constipation-related quality-of-life metrics [31]. Similarly, Konjac glucomannan, as a bioactive dietary fiber, also provided numerous health benefits such as controlling obesity and body weight, prebiotic benefits, constipation alleviation, reducing inflammation caused by intestinal related diseases, balancing gut microbiota, immune system modulator, and reducing the risk of colorectal cancer [32].

### 4.3. Synbiotics

Synbiotics are biological agents used in combination with probiotics and prebiotics and characterized by the simultaneous action of probiotics and prebiotics. Synbiotic intake can efficiently regulate gut microbiota and improve stool frequency, stool consistency, and constipation-related symptoms [33]. Besides, synbiotics consisting of fructooligosaccharide (FOS) and probiotics were used to intervene with constipation patients and also achieved similar results [34]. Increasing evidences indicated that both probiotics and prebiotics could increase the body's resistance to pathogenes and affect the body's immune system. Synbiotics may combine the effects of probiotics and prebiotics to improve gut microbiota, intestinal environment, and intestinal peristalsis and promote intestinal secretion, but all related studies did not achieve the desired results. The effects of synbiotics supplement (the combination of BB12, LP01, and inulin-oligofructose) on functional constipation symptoms among the respective individuals were explored [35]. The defecation frequency and stool type in the treatment group were improved significantly compared to those in the placebo group, but the differences in stool evacuation were not significant and synbiotics failed to demonstrate benefits over the controls due to the high placebo effect. The synergistic effect of probiotics and prebiotics in synbiotics had not been fully demonstrated. Hence, the actual role of synbiotics in constipation-associated gut microbiota still requires further studies based on better scientific design and larger samples.

### 4.4. Fecal Microbiota Transplantation (FMT)

Fecal microbiota transplantation (FMT) is the injection of donor feces into the gut to improve microbial diversity [36] and alleviate constipation. The evaluation study on the efficacy and safety of FMT on slow transit constipation (STC) indicated that FMT increased spontaneous bowel movements, reduced colon transit time, and improved PAC-SYM scores and PAC-QOL scores [37]. FMT could change the disordered fecal microbiota without significant side effects. GI symptoms, depression, anxiety, and sleep were improved [38]. After transplanting the fecal microbiota of constipation patients into mice, constipation occurred and SERT expression was significantly upregulated in colon tissue, but 5-HT levels were decreased. Besides, the intestinal flora abundance was changed, indicating that intestinal dysregulation might upregulate the expression of serotonin transporter (SERT) and then lead to the development of chronic constipation [39]. In addition to the application in constipation treatment, FMT can treat many other diseases, especially autism spectrum disorders [40], recurrent CDI (RCDI), severe CDI, and complex CDI. FMT has been proven to be a safe and effective treatment option [21].

## 5. Gut Microbiota and Chinese Medicine

Some Chinese scholars used CM to treat chronic constipation and achieved certain achievements. In the study on the mechanism of drug action, CM showed an effect on gut microbiota. Due to the current unsatisfactory effect of long-term use of laxatives, increasing scholars studied the efficacy and mechanism of CM to relieve constipation from the perspective of gut microbiota. There are many similarities between gut microbiota and CM theory, especially CM holistic concept, CM Yin-Yang balance theory, and CM constitutional theory.

### 5.1. Gut Microbiota and the Holistic Concept of Chinese Medicine

**Figure 3:**
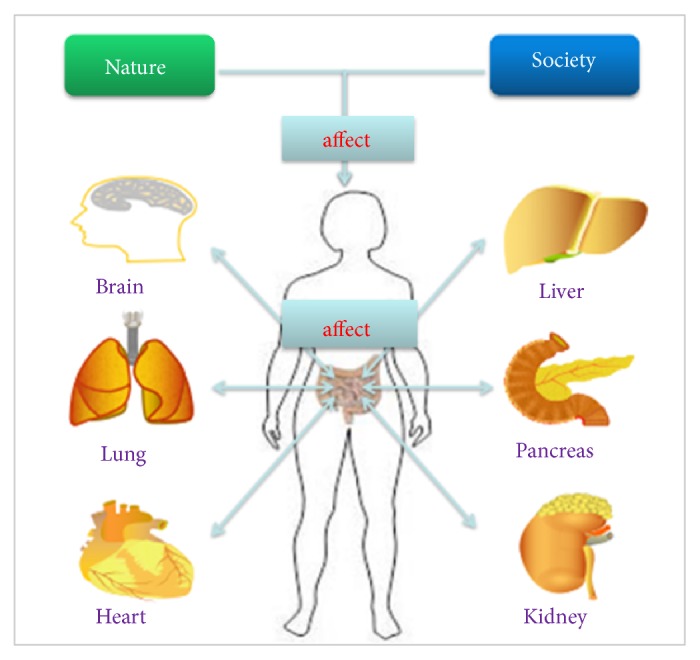
Gut microbiota and the holistic concept of CM. Natural and social environments can affect host gut microbiota and the gut microbiota interacts with host organs.

CM studies focus on the normal physiological activities and disease states of the human body from a holistic perspective. The holistic concept involves two parts. Firstly, natural and social environments can affect the structures and functions of human body. Secondly, the gut interacts with other organs (Figure 3).

First of all, human body is an integral part of the nature. According to CM theory, the changes in natural and social environments can affect the structures and functions of human body, including the gut microbiota. For instance, western diets containing less fibers are prone to lead to constipation [41]. In northwestern China with the dry climate, the incidence of constipation symptoms is growing [42]. Besides, the gut microbiota is involved in the formation of gut immunity, but the structure of the gut microbiota may also change and affect the gut immunity when the environment and climate change [43].

Secondly, according to the holistic concept of CM, organs, tissues, and cells constitute human body as the whole organic structure, and they are interconnected and interact with each other. Therefore, local changes may lead to the functional changes of the whole body. Gut is the largest digestive organ of the human body and is closely related to the life activities of human beings. In many studies [44–47] on the gut-liver axis and the gut-brain axis, the gut microbiota was treated as an important bridge between the gut and other organs.

There is a symbiotic, interdependent, and interacting relationship between the gut microbiota and the host/environment. Therefore, the study on the gut microbiota should focus on its connection with the host and environment.

### 5.2. Gut Microbiota and Yin-Yang Balance Theory of Chinese Medicine

**Figure 4:**
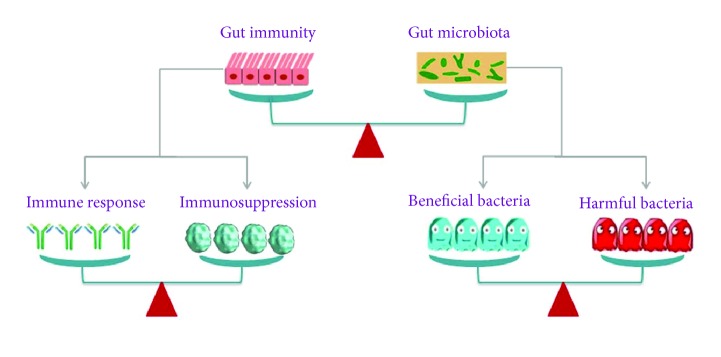
Gut microbiota and Yin-Yang balance theory of CM. The balance concept of CM emphasizes that the balance is the key to maintaining a healthy state, including the balance between gut immunity and gut microbiota, the balance between immune response and immunosuppression, and the balance among bacteria. Once one of the above balances is broken, the disorder may appear.

The philosophical foundation of CM theory stems from native Chinese ancient philosophy thoughts, which believe that Yin and Yang are a generalization of the relative attributes of related things or phenomena and the opposites of the same thing. Everything in nature contains two aspects of Yin and Yang, while Yin and Yang coexist in a whole system. Additionally, the Yin and Yang maintain the relative dynamic balance through symbiosis, interdependence, mutual inhibition, growth and decline, and mutual transformation [48]. It is worth noting that the gut microbiota is also in a state of dynamic equilibrium and is balanced by the body's immunity. The Yin-Yang balance between the host and the gut microbiota is manifested in three aspects: the balance between host's immunity and the gut microbiota, the balance among bacteria, and the balance between immune response and immune tolerance [49] (Figure 4). Since the birth, the microbiota colonizes the human gut and has a symbiotic relationship with the host. The gut microbiota and host immunity are interdependent and mutually restrictive. In addition, there is also a symbiotic and restrictive relationship among different gut microbes. The beneficial bacteria and the harmful bacteria compete with each other but coexist in the gut. In the pathological process of constipation, the balance mechanism among different gut microbes can effectively inhibit the growth of spoilage bacteria, improve the gut environment, and alleviate constipation symptoms. Contradictory relationships are beneficial for maintaining a dynamic balance between the human and the gut microbiota and maintaining gut homeostasis and health. Once one of the above balances is broken, the disease may occur [50]. Consequently, the unity and opposites of gut microecology have a commonality with the Yin and Yang theory of CM.

### 5.3. Gut Microbiota and the Chinese Medicine Constitutional Theory

**Figure 5:**
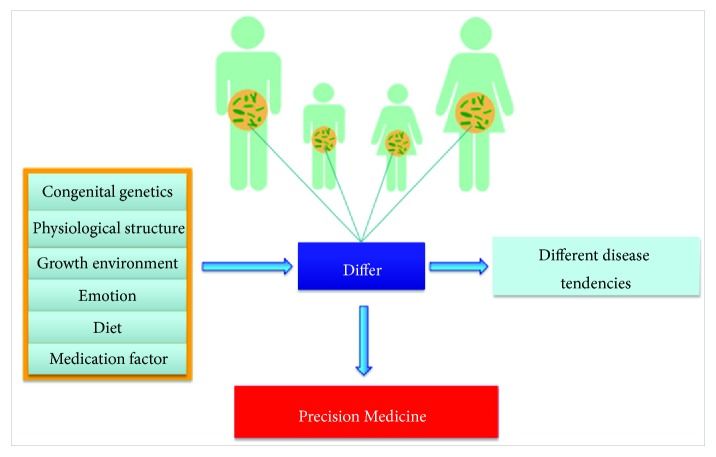
Gut microbiota and the CM constitutional theory. CM constitutional theory believes that a variety of factors lead to different gut microbiota among individuals, so the diseasing tendency will be different. Therefore, the precision medicine may be the future direction of development.

CM constitution is an objective existence in the life phenomenon. It is a comprehensive and relatively stable trait in the morphological structure, physiological function, and psychological state in the individual life process [51]. The CM constitutional theory holds that there are differences among normal individuals, and different individuals have their own particularities in physiology, function, and psychology. These particularities affect people's adaptation ability to the natural and social environment and their resistance to diseases as well as the susceptibility to certain pathogenic factors during the pathogenesis and the propensity for disease development during pathological processes [43]. The microbiota living in the gut affects the physiology and metabolism of the human body.  Figure 5 shows that the gut structure and function as well as the composition and abundance of gut microbiota are different among individuals due to congenital inheritance, growth environment, dietary habits, medication factors, personality emotions, and anatomical structures. Furthermore, the individual's body and immunity have an obvious discrepancy due to these differences. Accordingly, after the microbiota is dysregulated, different diseases occur such as diarrhea, constipation, and inflammatory bowel disease. Even if two different individuals have the same disease, different microbiota changes may occur and the composition of the original bacteria is not exactly the same. Therefore, in the future studies on gut microbiota, individual differences and precision medicine should be emphasized.

### 5.4. Relationship between Chinese Medicine and Gut Microbiota

In a new report in the journal* Science*, professor Zhao Liping lost 20 kilograms in 2 years after he adopted a regimen involving Chinese yam and bitter melon-fermented prebiotic foods, while his blood pressure, heart rate, and cholesterol levels declined. In this period, he found that* Faecalibacterium prausnitzii *increased from an undetectable level to 14.5% of his total gut bacteria [52].

In order to determine whether Gegen Qilian Decoction (GQD), a traditional Chinese herbal formula, can regulate the composition of the gut microbiota during the treatment of type 2 diabetes (T2D), 187 T2D patients were randomized to, respectively, receive high-dose GQD (*n* = 44), moderate GQD (*n* = 52), low-dose GQD (*n* = 50), or placebo (*n* = 41). The results showed that GQD treatment could enrich beneficial bacteria, such as* Faecalibacterium* spp. It indicated that the Chinese herbal formula GQD could affect the structure of the gut microbiota [53].

In 2018, Tong's team and Zhao's team collaborated on a multicenter, randomized, open-label clinical trial to treat patients with T2D and hyperlipidemia with metformin and Chinese herbal compound (AMC composed of 8 herbs, namely,* Rhizoma Anemarrhenae*,* Momordica charantia*,* Coptis chinensis*,* Salvia miltiorrhiza*, red yeast rice,* Aloe vera*,* Schisandra chinensis*, and dried ginger). The results showed that both drugs significantly alleviated hyperglycemia and hyperlipidemia and changed the gut microbiota in patients. Besides, the increase in coenriched bacteria represented by* Blautia* spp. was significantly associated with the improvements in lipid and glucose homeostasis. Importantly, the effect of AMC on the gut microbiota was greater and the improvements in homeostasis model assessment of insulin resistance (HOMA-IR) and plasma triglycerides were better due to the increase in coenriched bacteria represented by* Faecalibacterium* spp. [54].

All of the above studies have proven that Chinese medicine closely affected the gut microbiota. Xu J et al. summarized the molecular mechanisms of their interaction into three aspects. Firstly, the gut microbiota can convert compounds in Chinese medicine into metabolites with biological activity/toxicity. In addition, Chinese medicine compounds also improve the composition of gut microbiota, thereby improving dysfunction and related pathological conditions. Finally, the gut microbiota mediates the interactions among various chemical components in Chinese medicine [55].

### 5.5. Chinese Medicine Can Alleviate Constipation by Modulating Gut Microbiota

The relationship between Chinese medicine and chronic constipation has been extensively studied. Furthermore, some Chinese medicines have been fully proven to be able to improve chronic constipation.

#### 5.5.1. Effect of Chinese Medicine on Constipation

Certain herbs or herbal formulas have been widely used to treat chronic constipation in East Asia. Especially in China, more and more patients with chronic constipation are more willing to seek help from Chinese medicine. Furthermore, many studies proved that Chinese medicine was an effective treatment way for chronic constipation.

The patients with STC (45 cases) were treated for 3 weeks with Huangxin Runchang Pian, a Chinese patent drug made by Shuguang Hospital affiliated to Shanghai University of Traditional Chinese Medicine, and 45 cases in the control group were treated with Danggui Longhui Wan, a Chinese patent drug that had been applied in clinical practices. The total clinical effective rates after the treatment and in 1 month after stopping medication were, respectively, 95.6% and 82.2% in the treatment group and 93.3% and 80.0% in the control group. However, there was no significant difference between the two groups [56]. A randomized, double-blind, and multicenter study was conducted to confirm the efficacy and safety of Xiao'er Biantong Granules (XEBT), a new Chinese patent drug, in the treatment of chronic constipation in 480 children with FC. The mean frequencies of spontaneous bowel movements (SBM) in the XEBT group and the placebo group were, respectively, 8.89 and 5.63 and there were, respectively, 86.87% and 30.91% subjects with SBM ≥ 3/week in two groups. The difference between the two groups was significant. Besides, the XEBT group had a significant effect on median effective time of defecation, the main symptom score, and the rate of symptom disappearance, and the safety was good. XEBT had the better curative effect and good tolerance compared to the placebo in the children with functional constipation [57]. A meta-analysis of a randomized controlled trial for Chinese Herbal Formula, Modified Buzhong-Yiqi-Tang (MBYT), in the treatment of adult functional constipation showed that MBYT, which had been widely applied in the treatment of functional constipation, could significantly improve the constipation symptoms, compared to stimulant laxatives, prokinetic agents, osmotic laxatives, and biofeedback [58]. Tongbian Decoction, a combination of CM, could alleviate senile constipation due to the increase in SCFA and butyric acid in feces [59].

In order to clarify the mechanism of certain Chinese medicines in constipation treatment, more fundamental studies had been conducted. CM ingredients, such as polysaccharide saponins and flavonoids, interact with the gut microbiota to adjust its composition. For instance,* Dendrobium candidum* polysaccharide, the main component of Chinese herbal medicine* Dendrobium candidum*, is mainly composed of monosaccharides such as glucose, galactose, xylose, mannose, rhamnose, glucuronic acid, and galacturonic acid and has been proven to improve the gut environment and alleviate constipation by enhancing the body immunity and regulating gut microecological balance and gut enzyme activity [60].* Dendrobium officinale* Kimura et Migo is a traditional Chinese medicine and health food commonly used to promote body fluid production. Constipation mice showed a higher gastrointestinal transit rate and faster bowel movements after being given an ultrafine powder of* Dendrobium officinale *(UDO). In addition, UDO treatment significantly increased the levels of acetylcholinesterase (AChE), gastrin (gas), motilin (MTL), and substance P (SP) in serum, whereas the serum level of somatostatin (SS) was obviously decreased. Therefore, UDO exhibited a significant laxative effect, which might be related to the elevated levels of AChE, Gas, MTL, and SP and the reduced production of SS [61].* Cistanche tubulosa* is also a well-known Chinese medicine that can be used to treat constipation, especially senile constipation.* Cistanche tubulosa* extract (CTE) can relieve depression through the gut-brain axis by regulating gut microbiota and short-chain fatty acids [62]. Furthermore, Hanbing L et al. [63] found that* hemp seed* oil could facilitate defecation and relieve constipation.

In China, some Chinese medicine formulas are also particularly concerned due to their multiple-target and multipath mechanism. Liu D et al. [64] used Zengye Decoction (ZYD), a CM formula consisting of* Radix scrophulariae*,* Ophiopogon japonicus*, and* Radix rehman*n*ia*, to treat elderly constipation rats. The results indicated that ZYD exerted a therapeutic effect by restoring the gut microbiota of constipation rats and regulating host metabolites. In addition, Yangyin Runchang Decoction (YRD) consists of Zengye Decoction and Zhizhu pills are important Chinese medicine for the treatment of constipation. YRD had been proven to be effective for the treatment of STC mouse model by restoring the stem cell factor (SCF)/c-kit pathway, increasing interstitial cells of Cajal (ICC) counts and enhancing ICC function [65]. Moreover, lubricating gut pill (LGP), a Chinese medicine formula, had been used to treat constipation rats and LGP enhanced fluid and Cl^−^ secretion through prostaglandin receptor signaling as well as the cAMP and protein kinase A pathway [66].

#### 5.5.2. Effect of Chinese Medicine on Gut Microbiota in Constipation

In recent years, it had been confirmed that certain Chinese herbs or formulas could help regulate gut microbiota and maintain the stability of the gut environment in theoretical, clinical, or animal experimental studies. After the treatment with* Dendrobium candidum* polysaccharide, the total quantity of* E. coli* was restored significantly, indicating that* Dendrobium candidum* polysaccharide had a certain inhibitory effect on* Escherichia coli*. In addition,* Dendrobium candidum* polysaccharide had an obvious proliferative effect on* Lactobacillus* and* Bifidobacteria* [60]. However,* hemp seed* oil was found to affect the gut microbiota by adjusting the ratio of cecal thick wall bacteria/bacteroides and increasing the quantities of* Bifidobacterium* and* Clostridium butyricum*,* Lactobacillus*, and the levels of acetic acid and butyric acid [63]. CTE administration resulted in an increase in* Weissella*,* Bacteroides*, and* Arabidopsis*, a decrease in the quantity of* Ruminococcus*, and a significant decrease in the quantity of* Deinococcus*, which was significantly increased in the CUS model group. CTE could reverse the disordered concentrations of acetic acid and caproic acid to a reasonable level [62]. Slow transit constipation (STC) rat model was treated with aqueous extracts of* Herba Cistanche* (AEHC), which increased the protein and mRNA levels of c-kit and enhanced labeling of interstitial cells of Cajal (ICC). In addition, the level of SCF, a ligand of c-kit, was also regulated. The experiment result indicated that AEHC could improve the function of ICC via a signaling pathway involving SCF, c-kit, and PI3K and thereby enhance colonic motility [67]. Altogether,* Cistanche tubulosa* is a kind of* Herba Cistanche* and the extracts of* Herba Cistanche *can improve the function of ICC, enhance colonic motility, affect the structural composition of the gut microbiota, and reverse the disordered concentration of SCFA. Therefore, the gut microbiota may mediate the regulation role of the extracts of* Herba Cistanche* in the signaling pathway involving PI3K, SCF, and c-kit and improve the function of ICC and colonic motility.

Chinese medicine decoction can change the gut microbiota in constipation patients and treat gut dysbiosis. For instance, ZYD described above reduced the relative abundance of harmful bacteria, such as* Desulfovibrio*,* Prevotella*,* Ruminococcus*, and* Dorea*, and increased the abundance of* Oxalobacter*,* Clostridium*, and* Roseburia* [64]. In addition, ZYD regulated carbohydrates, SCFA, amino acids, and amines. ZYD treatment increased the energy reserve, enhanced the function of glutathione, regulated amino acid metabolism, inhibited methane metabolism, and reduced bacterial toxins. These results indicated that ZYD regulated the gut microbiota of constipation rats and altered the host's endogenous metabolites through the gut microbiota to achieve effects [64]. Tongbian Decoction had a mitigation effect on senile constipation by increasing SCFA and butyric acid in feces, but its regulating effect on the type or quantity of gut microbiota had not been studied [59]. Another CM formula, Jieduquyuziyin Prescription, could effectively restored gut microbiota of systemic lupus erythematosus (SLE) mice and regulated the balance of metabolites [68]. YRD also significantly increased the number of ICC and improved the function of ICC, but its relationship with gut microbiota was still unclear and deserved further study [65].

In short, the holistic view is an important concept for CM. Climate, environment, living habits, diets, and emotions can all affect the composition and function of gut microbiota. Firstly, organs, tissues, and cells of human body are also interrelated and interdependent. The gut microbiota is closely related to multiple organs through the gut-liver axis and the gut-brain axis. The relationship between the gut microbiota and other organs or tissues deserves further study. Secondly, the balance of Yin and Yang is also a core point of view for CM. The interaction between the gut microbiota and human immunity is balanced so that pathogen infection or allergic reaction does not occur. The balance between beneficial bacteria and harmful bacteria can maintain the stability of the intestinal environment and normal intestinal function and avoid constipation symptoms. Therefore, a single supplement of a certain bacteria may not achieve the desired effect. Conversely, restoring the proportion and abundance of the gut microbiota to a normal state may be the valuable idea in the future. Thirdly, the CM constitutional theory focuses on the differences among individuals. The physical strengths of individuals are different, and the compositions of the gut microbiota are not completely the same among individuals. Therefore, the individualization is emphasized during the treatment and it is consistent with the thinking of “Precision Medicine.” In summary, the studies on the gut microbial treatment of constipation inspire us since gut microbiota has individual differences. The microbial treatment should be carefully selected based on the microbiota of constipation patients and then supplement targeted probiotics, prebiotics, and synbiotics for constipation patients. In addition, treatment constipation by Chinese medicine is a potential therapy. Chinese medicine can restore the normal composition and function of the gut microbiota, regulate endogenous metabolites, improve the gut environment and function, and relieve symptoms caused by constipation.

## 6. Summary

Collectively, chronic constipation is a gastrointestinal disease with a high incidence of gut microbiota, which plays a crucial role in the pathogenesis of chronic constipation. Gut microbiota and its metabolites affect disease pathogenesis and pathological processes in a variety of ways, including gut digestion and absorption, gastrointestinal motility and secretion, gut immune system activation and inflammation, and gut-brain axis. As mentioned above, the main features of gut microbiota alteration in patients with chronic constipation are the relative reduction of beneficial bacteria such as lactobacilli and bifidobacteria and the relative increase in potential pathogens. Recently, growing clinical and experimental studies indicated that the microbial treatment methods for patients with chronic constipation, such as probiotics, prebiotics, and fecal transplants, demonstrated the possibility of alleviating chronic constipation from the perspective of the gut microbiota. However, its safety and effectiveness should be verified by further studies. Interestingly, CM has a positive impact on constipation improvements. Some CM herbs and formulas can also help regulate gut microbiota and maintain the stability of gut microecology, but the mechanism of action of CM monomer drugs or Chinese medicine compounds has been seldom reported and whether the gut microbiota mediates CM and improves constipation still requires further studies. In conclusion, CM is a great treasury of China and it is well known for its multiple pathways, multiple targets, and few side effects and therefore is worth further exploration. Additionally, the influence of the gut microbiota on the pathophysiological mechanism of chronic constipation is not completely clear, so it is necessary to further study the mechanisms of the gut microbiota on the pathophysiology of the gastrointestinal tract. It is significant to study treatment methods to restore the gastrointestinal microbial environment.
